# The Development of the DDads Questionnaire: Awareness, Knowledge and Attitudes of the General Population Towards Paternal Depression

**DOI:** 10.3389/fpsyt.2020.561954

**Published:** 2021-01-21

**Authors:** Joeri Vermeulen, Ronald Buyl, Florence D'haenens, Dennis Demedts, Sandra Tricas-Sauras, Ihsane Haddani, Maaike Fobelets

**Affiliations:** ^1^Department Health Care, Erasmus Brussels University of Applied Sciences and Arts, Brussels, Belgium; ^2^Department of Public Health, Biostatistics and Medical Informatics Research Group, Faculty of Medicine and Pharmacy, Vrije Universiteit Brussel, Brussels, Belgium; ^3^Department of Public Health, Mental Health and Wellbeing Research Group, Faculty of Medicine and Pharmacy, Vrije Universiteit Brussel, Brussels, Belgium; ^4^Public Health School, CR5 - Department of Social Approaches to Health (CRISS), Université Libre de Bruxelles, Brussels, Belgium

**Keywords:** questionnaire development, validation study, paternal depression, perinatal mental health, fathers, depression, mental health

## Abstract

**Objectives:** Paternal perinatal depression affects ~10% of new fathers and is known to have a negative impact on men's relationship with their partner as well as with their baby. The attitudes of the general population toward paternal depression have received scant attention in the scientific literature. A better understanding of paternal depression might improve the health literacy of the population and also assist professionals and policy makers to adequately address this issue, to ultimately refine the existing health care alternatives for them. This paper describes the Belgian development, face and content validation of the DDads (Depression in Dads) questionnaire. Its focus is to identify the awareness, knowledge and attitudes of the general population toward paternal perinatal depression.

**Study Design:** The DDads was developed using a three-step model with the following phases: (1) identification of the content domain, (2) item generation and (3) construction of the questionnaire. For the DDads validation a (a) Delphi method with content experts (*n* = 17) and (b) a cognitive debriefing method with lay experts (*n* = 20) were used to assess the clarity, relevance, wording and layout.

**Results:** The questionnaire consists of three main components comprising: (1) three questions on awareness, (2) three questions on knowledge and (3) one question on attitudes and beliefs. After round one validation, all questions were considered content valid for relevance (I-CVI 0.94–1.00), and six questions for clarity (I-CVI 0.65–1.00). Scale content (S-CVI/Ave 0.93) and face validity (Face Validity Index 1.00) was obtained. One question was revised and split into two questions in a second round. For one of these questions, item content (0.80–0.93), scale content (0.92) and face validity (1.00) was reached. The one question, exploring the causes of paternal perinatal depression, remained inappropriate and was removed from the DDads. One last question was removed after interviews with lay experts.

**Conclusions:** We developed an instrument to establish awareness, knowledge and attitudes of the general population toward paternal perinatal depression in Belgium. The DDads can be valuable in identifying knowledge gaps. It can help to inform policy makers and health professionals to identify gaps and predisposed attitudes in society toward paternal depression which may hinder appropriate management.

## Introduction

Perinatal mental health refers to mental health during pregnancy until 1 year after childbirth (1). It has been described as a time of increased risk for developing depression in both women and men (2–4). There is growing evidence suggesting that pregnancy and childbirth may provoke depressive symptoms, not exclusively in women but likewise in men (5). Paternal perinatal depression involves the occurrence of depressive symptoms in men in either the pre-natal or post-partum period (6).

Knowledge about the prevalence of paternal antenatal and post-partum depression is limited (7), but it is estimated that the prevalence of paternal perinatal depression during pregnancy until 1 year after childbirth is ~5–10% (3, 8). Recent Irish data suggested prevalence values of paternal postnatal depression of a 12% (9). According to a recent meta-analysis, the prevalence of paternal antenatal (9.76%) and that of post-partum depression (8.75%) is common. Europe has the lowest reported prevalence of paternal post-partum depression (5.52%), followed by the United States (9.43%) while the highest rates are reported in the Western Pacific (10.06%) (7). Rates of paternal post-partum depression are varied across different countries and continents, which might be explained by cultural preconceptions, e.g., social acceptance of mental health problems, differing interpretations of depressive symptoms or diverse expectations with respect to paternal infant care and its responsibilities (10). The variation of these figures can be attributed to various factors, such as the heterogeneity of assessment methods, biased translations of instruments, the study location, the publication year and the presence of maternal depression (6).

One of the strongest risk factors for the development of depressive symptoms in fathers is maternal perinatal depression (1, 11). In fact, when the female partner suffers from depression, the prevalence of paternal perinatal depression can be as high as 24–50% (12). A recent Irish study observed risk factors not previously reported (9). Some of those included: not having paternity leave or men whose partners were cared for in the public healthcare system. Women and men both express and manage their depressive symptoms differently. While maternal perinatal depression is characterized by a depressed mood, anxiety, feelings of inadequacy, loss of control, inability to cope, fatigue, and despair (4), paternal perinatal depression is characterized by additional symptoms (10). It is well-documented that men are more likely to display a hyperactive behavior, irritability, anger and may have lower control over their impulses. Depression in men may in addition be masked by somatic complaints, avoidance behavior, interpersonal conflict, and drug and/or alcohol use (11). The so-called “masked men's depression,” may include symptoms as: irritability, rage, emotional rigidity, and sleep disorders (10). Paternal perinatal depression is often under-assessed or undiagnosed because of these indefinite clinical features (13).

Paternal perinatal depression effects not only the relationship between partners, it is also related to emotional, behavioral, and developmental effects on children (1). Children of fathers with depressive symptoms face increased risks of adverse emotional and behavioral outcomes (1, 14). Promoting paternal psychological well-being, and preventing and treating paternal perinatal depression may therefore benefit the whole family (15).

Despite the existing body of knowledge on paternal perinatal depression and the importance of paternal mental health to family functioning, the research on evidence-based interventions for paternal depression is limited (2). As the prevalence of perinatal depression in men is relatively high, effective prevention, regular screening, and appropriate treatment need to be implemented (7). A recent review of 63 articles (16), revealed that routine screening and assessment of both partners across the perinatal period is strongly suggested. More attention needs to be paid to the mental health of fathers during the perinatal period, as fathers are underscreened, underdiagnosed and undertreated for paternal perinatal depression (9).

Men with depressive symptoms may feel excluded by health professionals and not understood by their environment, as fathers are mainly expected to push their own concerns aside in early parenthood (17). The attitudes of the general population toward paternal depression have traditionally received scant attention in the scientific literature. A survey to assess the health literacy of the Australian population on maternal postnatal depression showed high rates of awareness toward postnatal depression whereas, antenatal anxiety and antenatal depression are unknown to the public. Another Australian study using a combination of custom-designed questions on maternal postnatal depression showed positive results in recognizing maternal post-natal depression (18). A third Australian study using case-vignette only showed a high ability of the population to recognize maternal postnatal depression (19). Finally, a recent study assessing the mental health literacy of maternal and paternal post-partum depression, showed a higher symptom recognition of postnatal depression for women, which again highlights the need for an increased awareness of paternal perinatal depression (20). Therefore, a better insight in the health literacy of the general population concerning paternal depression could ultimately inform health professionals and policy makers on: how to adequately address these men and how to better use the existing health care options for men with perinatal depression. This paper describes the development of DDads (Depression in Dads) and its face and content validation. The ultimate purpose of DDads is to establish awareness, knowledge and attitudes of the general population toward paternal depression.

## Methods

### Description of the DDads Questionnaire

#### Development of the DDads Questionnaire

The DDads instrument was developed in three steps, as described by Zamanzadeh et al. (21). As such (1) a comprehensive literature review was conducted in order to identify the content domain, (2) the instrument items were generated based on the literature review and the teams' expertise, and (3) the DDads team constructed the instrument entirely.

The DDads questionnaire is based on several pre-existing tools: an existing questionnaire by Highet et al. focusing on maternal depression (22), a literature review on paternal post-partum depression (14), and the Diagnostic and Statistical Manual of Mental Disorders (DSM)-5 (2013) criteria (23). While all the questions of the questionnaire were developed and put together in English and once the final questions were selected, the forward/backward translation was applied (English to Dutch, JV, FD, DD, MF–Dutch to English, ST, JV, MF), to identify discrepancies between both versions, as is recommended to obtain reliable results and also necessary for validation for the local context (24). The final Dutch version was agreed on by the research team, and the design of the questionnaire was established.

Ethical approval was obtained from the University Hospital Brussels and the Vrije Universiteit Brussel (VUB), Belgium in April 2019 (registration number: B.U.N./143/201/939/907).

### Design

**Figure 1:**
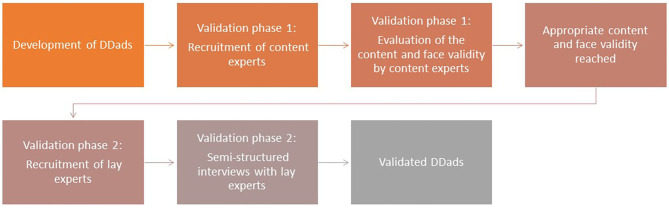
Flowchart of recruitment of participants and data collection.

As suggested by the literature (21, 25, 26), a two-phase validation study was set-up for the construction of the DDads tool including:

1. a Delphi study with experts focusing on the content (content experts)
2. a cognitive debriefing method with lay experts (Figure 1).

Content experts were professionals with work or research experience in the domain of interest, while lay experts were the potential study subjects (21), representing the population for whom the instrument is being developed. A Delphi method was chosen to perform a first validation on content and face validity. As reiterated by the scientific literature, this method is advised for achieving consensus on issues where none or little information previously existed. The Delphi method process that gathers information in a structured way, in a series of consecutive rounds until consensus is reached (27). A cognitive debriefing method was used to involve the lay experts. Their verbal open feedback was asked on each question regarding clarity, relevance, wording and layout and to make suggestions (28).

### Participants

#### Phase One: Content Experts

An expert panel was invited to assess the content of the DDads. Currently, there is no standardized way to calculate the appropriate sample size for expert-consensus in Delphi studies (29). However, a minimum sample size of ten experts is considered adequate (28), we intended to include fifteen content experts (21), all recruited based on their expertise in the perinatal mental health domain in Belgium. Maximum variation sampling was used (30), including experts from different fields of expertise: education, clinical, and governmental disciplines were recruited.

Possible content experts were invited by a personal e-mail in June 2019. The invitation included information about the study, the team and an informed consent form as well as a specific link to the DDads survey site. Participation to this study was voluntary.

From the 21 initially invited content experts contacted, a total of 17 experts agreed to participate in the validation process. Experts from the medical, nursing and midwifery fields were included. They were related to professional organizations/regulatory bodies (*n* = 2), research (*n* = 5), primary care (*n* = 2), practitioners in the clinical midwifery/nursing in the perinatal domain (*n* = 2), clinical midwifery/nursing in perinatal mental health (*n* = 2), and education (*n* = 6). Additionally, an obstetrician, a psychologist, a psychiatrist, a neonatologist and, an occupational therapist participated. Experts could be related to more than one domain.

#### Phase Two: Lay Experts

Inclusion criteria for lay experts included: both men and women above the age of 18 years, native Dutch speakers, from all educational levels (i.e., primary, secondary, and tertiary educational level). As such, they shared the characteristics of the potential study subjects.

Participants were recruited using convenience sampling. Possible candidates were randomly addressed by a researcher [IH] in a shopping mall and a train station in a municipality in Belgium (Dutch speaking part) in January 2020. Candidates were informed about the study and asked to participate. If they expressed their interest in our study, lay experts were asked to read the information letter and provide their consent. Socio-demographic information was also collected as recommended (28).

A total of 28 potential participants, women and men, were approached to participate, eight of which were not able to partake due to lack of time. In total, ten women and ten men (age range between 18 and 65 years old) with educational levels varying from primary education to tertiary education and differences in parity agreed to participate (Table 1).

**Table 1:** Characteristics of the lay experts.

**Lay expert ID**	**Age (years)**			**Educational level (highest completed education)**			**Parity**		
	**18–35 (*n* = 12)**	**36–55(*n* = 6)**	**≥56 (*n* = 2)**	**No education/Primary education only (*n* = 2)**	**Secondary education (*n* = 6)**	**Tertiary education (*n* = 12)**	**0 (*n* = 5)**	**1–2 (*n* = 9)**	**≥3 (*n* = 6)**
1	X					X		X	
2		X				X			X
3		X			X			X	
4	X					X		X	
5	X				X			X	
6		X				X			X
7	X				X		X		
8	X				X		X		
9			X	X					X
10	X				X			X	
11		X				X			X
12	X					X	X		
13	X					X	X		
14			X	X				X	
15	X					X	X		
16		X			X				X
17	X					X		X	
18	X					X		X	
19		X				X			X
20	X					X		X	

### Evaluating Content and Face Validity

Content validity, defined as “the degree to which an instrument has an appropriate sample of items for the construct being measured” (30) is a requirement for construct and criterion-related validity. Face validity indicates that the instrument appears to be valid “on its face” (28). It refers to whether the designed instrument is apparently related to the construct underlying the study, whether experts agree with the items and wording used to accomplish the aims of the research. Face validity is necessary for any instrument as it increases its acceptance by potential users (31).

Content and face validity are obtained when both the lay and content experts acknowledge that the scale is appropriate for measuring pertinent attributes.

### Data Collection

#### Phase One: Evaluation of Content and Face Validity by Content Experts

The Delphi study involved an online survey–LimeSurvey GmbH (33), with brief information and instructions for the assessment of items. The first section included professional information of the participant, while the second section gathered feedback on the relevance and clarity of the items in terms of content and face validity. During this evaluation, a 4-level Likert rating scale was used by the experts. Content experts were instructed to assess every item's clarity on how clearly it was worded, while its relevance referred to how relevant it seemed for the research objectives. As suggested by the literature, responses ranged from: 1 = not relevant/not clear, 2 = somewhat relevant/somewhat clear, 3 = quite relevant/quite clear, 4 = very relevant/very clear (36). Experts assessed clarity and relevance consecutively on the same scale, and were encouraged to assess each item completely (28). The content validity was quantitatively measured by establishing the proportion of experts agreeing on the relevance and clarity of the items (35).

Regarding face validity, content experts were asked to assess whether items were appropriately worded to achieve the aims of the research (21). For instance, by using the following statement: “The instrument attains the research objectives,” could be rated 1 = strongly disagree, 2 = disagree, 3 = agree, 4 = strongly agree. Also, experts could suggest additional items and make comments.

The responses of individual content experts were only known by one researcher [MF] and remained unknown to the other experts and the rest of the research team. That particular researcher was thus able to encourage non-responders and follow up the process, as is suggested in literature (27). All information was anonymized during the analysis and combined in the final reports. Data was stored on a secured server located at the Vrije Universiteit Brussel in compliance with data management and the General Data Protection Regulation (2018).

In validation, carefully controlled feedback, to progressively seek consensus, is an iterative process. Opinions are commonly produced in the first round, in which experts may put forward additional ideas and draw on existing information. These ideas were returned to the experts in the consecutive rounds (37).

#### Phase Two: Evaluation of Face Validity by Lay Experts

After appropriate content and face validity of the DDads was achieved, lay experts were invited to assess the questionnaire using the cognitive debriefing methodology. Lay experts were asked to complete the questionnaire and their verbal open feedback was asked by the researcher [IH] on each question regarding clarity, relevance, wording and layout and to make suggestions (28). If necessary, further clarifications were evoked and attention was given to non-verbal signals.

### Data Analysis

#### Phase One: Evaluation of the Content and Face Validity by the Content Expert Panel

Two content validity indexes were calculated (CVIs): the content validity of the overall scale and that of individual items (36). To determine the CVI for each items' relevance and clarity (I-CVI), the number of experts assessing it as relevant or clear (rating 3 or 4) is divided by the total numbers of experts. The I-CVI expresses thus the degree of consensus between experts, with a value between 0 and 1.00. These values were interpreted as recommended in literature (21): if the I-CVI was higher than 0.79, the item was considered appropriate, and if the I-CVI was between 0.70 and 0.79 it needed revision. In that case the item was adapted based on the content experts' advice and subsequently included in a next Delphi round. If the I-CVI was below 0.70, the item would have to be removed.

The scale-level CVI (S-CVI), which is described as the proportion of total items considered to have content validity (34). The S-CVI was computed using the “average proportion of items rated as 3 or 4 across the different experts” (hereafter referred to as S-CVI/Ave), as recommend by Polit and Beck. (38). It has been suggested that the S-CVI/Ave should be conceptualized as the average I-CVI value, because this value focuses on average item quality rather than average performance by the experts (32). As is indicated in scientific literature (30, 32, 39), a S-CVI/Ave of ≥0.90 reflects content validity of the entire instrument.

In addition to the CVI, the face validity index was computed. The number of content experts who “agreed” or “strongly agreed” (i.e., rating 3 or 4) to the statement “The instrument attains the research objectives” was divided by the total number of experts. As suggested by the scientific literature (40), a face validity index of ≥0.80 demonstrates that the scale obtained face validity.

#### Phase Two: Evaluation of Face Validity by Lay Experts

The cognitive debriefing method was used mainly as a think aloud method, avoiding interviewer bias and minimal training requirements from the interviewer. The respondents were instructed prior to completion of the instrument to think aloud as he/she answers the questions. This data could be interpreted later in the context of comprehension, decision process and other aspects of pilot testing (26). Lay-experts were interviewed until data saturation was reached (41). To refine the analysis, the data and interpretations were later presented and discussed among the interviewer [IH] and the rest of the research team.

## Results

### Development of the DDads Questionnaire

The DDads instrument consists of three main components with a total of seven questions. Those are briefly presented next:

Component (1): “Awareness”: two open-ended questions with a maximum of four answers options possible and one open question regarding the occurrence of paternal depression. This section focuses on respondents' awareness about mental-health problems that men can experience during their partner's pregnancy and the first year after the childbirth.

Component (2) “Knowledge”: one multiple-choice question (9 items) with multiple answer options. This section focuses on causes and symptoms of depression and potential treatment avenues. One multiple-choice question (16 items) with multiple answer response options regarding treatment options and one multiple-choice question (11 items) with one answer option only about who to address, in case of depressed feelings.

Component (3) “Attitudes and beliefs”: this section considers common viewpoints and knowledge on the explored matter. One multiple-choice question (21 statements about paternal depression) with multiple answer options.

### Phase One: Evaluation of Content and Face Validity by Content Experts

#### Item Content Validity Index I-CVI

The first round confirmed that all seven questions were content valid for relevance (I-CVI 0.94–1.00), and 6 out of the 7 questions were content valid for clarity (I-CVI 0.65–1.00). One question, “What are the causes and symptoms of paternal perinatal depression?” was considered inappropriate for clarity (0.65). Although the I-CVI for clarity was below the threshold of 0.70 the research team decided to modify the question based on the experts' recommendations. Because this was a double-barreled question, it was revised and split into two questions and was included in the next validation round (Table 2).

**Table 2:** Item-content validity index I-CVI after round one.

	**Relevance**					**Clarity**				
**Questions**	**Received answers (total *n* = 17) *n***	**Relevant (rating 3 or 4) *n***	**Not relevant (rating 1 or 2) *n***	**I-CVI relevance**	**Interpretation**	**Received answers (total *n* = 17) *n***	**Clear (rating 3 or 4) *n***	**Not clear (rating 1 or 2) *n***	**I-CVI clarity**	**Interpretation**
In your opinion, what do you think are the most important mental health-problems that men experience during the pregnancy of their partners?	17	17	0	1.00	Appropriate question	17	15	2	0.88	Appropriate question
In your opinion, what do you think are the most important mental-health problems that men experience during the first year after the birth of their children?	17	17	0	1.00	Appropriate question	17	16	1	0.94	Appropriate question
What is the occurrence of paternal depression during the pregnancy of their partner and during the first year after the birth of their children?	17	16	1	0.94	Appropriate question	17	14	3	0.82	Appropriate question
What are causes and symptoms of paternal depression?	17	17	0	1.00	Appropriate question	17	11	6	0.65^*^	Inappropriate question
Which types of treatment are appropriate for men with a paternal depression?	17	17	0	1.00	Appropriate question	17	14	3	0.82	Appropriate question
If you (or your partner) would suffer from a paternal depression, who would you address to as first choice? Who would you prefer/advice to address as first choice?	17	17	0	1.00	Appropriate question	17	16	1	0.94	Appropriate question
Attitudes and believes regarding paternal depression	17	17	0	1.00	Appropriate question	17	17	0	1.00	Appropriate question

* *I-CVI < 0.70*.

In the second round (October 2019), 15 of the 17 content experts participated, still an acceptable variation sampling was achieved. The neonatologist, an educator and the occupational therapist withdrew from the second round.

The question considered as inappropriate regarding the causes and symptoms of paternal perinatal depression, was again revised as advised by the Delphi panel. One of these questions, “How can you recognize paternal perinatal depression (symptoms)?,” reached an acceptable item content validity for relevance (0.93) and clarity (0.80) in the second validation round. The second question, “what are the causes of paternal perinatal depression,” remained inappropriate, item content validity for relevance (1.00) and clarity (0.60), and was then removed from the DDads questionnaire (Table 3).

**Table 3:** Item-content validity index I-CVI after round two.

	**Relevance**					**Clarity**				
**Questions**	**Received answers (total *n* = 17) *n***	**Relevant (rating 3 or 4) *n***	**Not relevant (rating 1 or 2) *n***	**I-CVI relevance**	**Interpretation**	**Received answers (total *n* = 17) n**	**Clear (rating 3 or 4) *n***	**Not clear (rating 1 or 2) *n***	**I-CVI clarity**	**Interpretation**
How can you recognize a paternal depression (symptoms)?	15	14	1	0.93	Appropriate question	15	12	3	0.80	Appropriate question
What are the causes of paternal depression?	15	15	0	1.00	Appropriate question	15	9	6	0.60^*^	Inappropriate question

* *I-CVI < 0.70*.

#### Scale Content Validity Index S-CVI/Ave

The S-CVI/Ave, defined as the “average proportion of items rated as 3 (quite relevant/quite clear) or 4 (very relevant/very clear) across the various experts” (21), was 0.93. Nevertheless, the amendments made after the Delphi round one, the S-CVI/Ave remained appropriate; 0.92 which confirms content validity of the entire DDads questionnaire.

#### Face Validity Index

To determine face validity, content experts were invited to specify if items and wording of the questionnaire were appropriate for the aims of the research to be realized. Face validity was obtained after the first round, with a rating of 1.00. After a second round, the face validity remained 1.00 (Table 4).

**Table 4:** Face validity index.

	**Received answers**	**Relevant (rating 3 or 4) *n***	**Not relevant (rating 1 or 2) *n***	**Face validity index**	**Interpretation**
Does the instrument realizes the research objectives (round 1)	17	17	0	1.00	Appropriate
Does the instrument realizes the research objectives (round 2)	15	15	0	1.00	Appropriate

#### Suggestions From the Content Experts

Content experts were invited to make suggestions for improvement of the DDads or new items to be added. Most comments were related to wording and content and considered questions with an acceptable I-CVI for clarity >0.70 and I-CVI for relevance >0.70, nevertheless all suggestions were reviewed by the research team [JV, FD, DD, ST, MF]. After a consensus had been reached, the amendments (i.e., rewording and supplementary items in multiple-choice questions) were included in the second Delphi round.

### Phase Two: Evaluation of Face Validity by Lay Experts

#### Evaluation From the Lay Experts

The DDads took between 10 and 40 min to complete (median 20 min), which experts found acceptable. The lay experts praised the DDads for its comprehensiveness, structure and the logical sequence of the questions. Most of the lay experts (*n* = 16) did not know that paternal depression existed. Only four lay experts (who had a higher level of education according to demographic data) were aware of the existence of paternal perinatal depression.

Minor suggestions to improve its readability and usability were taken into account by the research team. The question exploring respondents' knowledge about the prevalence of paternal perinatal depression was considered as wide of the mark, and was removed as half of the lay experts mentioned they had to guess their answer on that question. Data saturation was obtained after twenty interviews, when no new themes emerged from the interviews.

The validated DDads questionnaire comprises three components as presented earlier with a total of six questions in its final version:

Component (1) “Awareness”: two open-ended questions with a maximum of four answer options for each question,

Component (2) “Knowledge”: two multiple-choice questions with multiple and one multiple-choice question with one answer option only,

Component (3) “Attitudes and beliefs”: one question to rate responder's agreement using a Likert scale on 21 attitudes and beliefs statements about paternal depression.

## Discussion

Our team developed a valid instrument (DDads) to determine the awareness, knowledge and attitudes of the general population toward paternal perinatal depression. In accordance with the 2 to 4 rounds usually required as suggested by Keeney et al. (27), the DDads questionnaire achieved both content and face validity after two Delphi rounds.

A question concerning the origins and symptoms of paternal perinatal depression was included on the first round. This question was then split into two questions because this was a double-barreled question in the second round. The question on the symptoms was withheld in our questionnaire since the question focusing on the causes was left out due to its low I-CVI on clarity. The question exploring respondents' knowledge about the prevalence of paternal perinatal depression was considered as wide of the mark. The lay-experts did not have any idea about the prevalence numbers and indicated they were gambling and therefore this question was removed.

Hitherto, published studies on health literacy focusing on maternal post-partum depression achieved high rates of awareness (18–20, 22). The study of Swami et al. evaluating mental health literacy of postnatal depression in women and men, suggested that less attention is paid to the recognition of paternal postnatal depression compared to maternal postnatal depression (20). As mentioned earlier, we left out a question regarding the prevalence of paternal perinatal depression, which had already suggest low health literacy levels of the general population on this respect. In addition, during the composition of the Delphi-panel, we faced difficulties in finding content experts in the field of paternal perinatal depression. We noticed that experts in paternal perinatal depression appear to be limited and expertise is centralized in a few specific clinical settings. Nevertheless, we were able to include experts of two out of the three specialized clinical settings existing in the Dutch speaking part of Belgium.

We consider that the DDads questionnaire can be very valuable in identifying knowledge gaps. This may subsequently inform health professionals and policy makers in due course which is essential to identify specific needs in our context. For instance, how to hypothetically identify gaps and biased attitudes in society toward paternal depression. In the end, the DDads may help initiate cross-cultural research in the domain of perinatal mental health, and make paternal depression visible and also debatable in society. We hope we can contribute to paternal perinatal depression recognition and facilitate a more family-centered approach in healthcare.

Some limitations faced by our study should be addressed. The DDads refers to perinatal depression during pregnancy until 1 year after childbirth. Besides two questions in the component “Awareness,” the DDads does not differentiate between antenatal and post-partum depression in men. As the assessment from the experts could considered somehow subjective, our study is susceptible to a possible bias due to our experts' sample (25). We acknowledge that Delphi studies, as a group consensus representing expert opinion rather than indisputable facts, have its limitations (27, 37). Nevertheless, we carefully enforced methodological rigor into our process to make sure that our process was robust throughout and we estimate that results were accurate. In this sense, the Delphi method [widely accepted in health research (27)], might not necessarily identify all possible options and some content have been missed. In addition, lay experts' opinion on the DDads' clarity, wording and layout were considered during the validation process.

Surveying the real condition of the healthcare system might provide challenges for the design of healthcare and provide guidance on the development and implementation of screening methods and concepts in healthcare. Based on our study findings, additional studies should address the standardization and measurement of existing tools to identify paternal perinatal depression along with specific interventions for men (10).

In this study content and face validity of the DDads was obtained, other types of validity such as construct validity and criterion validity and reliability e.g., test-retest reliability and internal consistency were not assessed. Future research is needed to establish further reliability and validity. We would like to conduct further data collection to assess the knowledge, attitudes and awareness in the general population in our setting. Also, to assess whether DDads could also be used to assess these attributes amongst healthcare professionals in specific settings.

In light of the scarce existing literature, further research is recommended to assess the experiences of fathers exploring aspects such as number of children, adopted children and the role of culture on the experience of paternal perinatal depression (16). Future research should involve the development of a robust screening tool which enables screening for paternal birth related anxieties and concerns (2).

Health professionals and authorities are recommended to be more vigilant to the early recognition of antenatal and post-partum paternal depressive symptoms, so that subsequent effective treatments can be implemented (7). In this sense, healthcare services should offer a wider array of services, such as offering tailor-made individual, couple and peer support groups to accompany pregnancy. But also, the needs of expectant fathers should be considered since as previously noted, this could facilitate the pregnancy process and have a further positive effect in the whole family (13).

The growing demand for cross-cultural comparisons in health care and the use of culturally adapted and valid scales is a contemporary subject (42). Rigorous forward and backward translation and validation for the local context is required to obtain reliable results (25). The evaluation of the use of DDads in other countries is therefore highly recommended for future cross-cultural research (43).

## Conclusion

We developed an instrument to establish awareness, knowledge and attitudes in the general population toward paternal depression in Belgium. The DDads appears useful in identifying knowledge gaps concerning knowledge and attitudes toward depression.

Our tool appears relevant for the use of health professionals and eventually policy makers toward the identification of specific needs of this group of the population. It is our hope that those needs are in the long run translated into tailored actions.

We ultimately hope to contribute to addressing biased societal attitudes toward paternal depression and to raise awareness of the topic for a better understanding and subsequent management. To conclude, the DDads may initiate cross-cultural research in the domain of perinatal mental health and make paternal depression visible and a subject of debate. We consider this to be essential toward its recognition and subsequently facilitate a more family-centered approach in healthcare, particularly in the cited context.

## Data Availability Statement

The raw data supporting the conclusions of this article will be made available by the authors, without undue reservation.

## Ethics Statement

The studies involving human participants were reviewed and approved by University Hospital Brussels and the Vrije Universiteit Brussel (VUB), Belgium in April 2019 (registration number: B.U.N./143/201/939/907). The participants provided their written informed consent to participate in this study.

## Author Contributions

JV, FD, DD, ST-S, and MF developed the conception and design of the study. MF and IH collected the data. MF and RB performed the statistical analyses. JV, RB, FD, DD, and ST-S supervised the development of the study design and data analyses. JV and MF wrote the first draft of the manuscript. JV, FD, RB, DD, ST-S, and MF contributed to manuscript revision and all approved the final submitted version.

## Conflict of Interest

The authors declare that the research was conducted in the absence of any commercial or financial relationships that could be construed as a potential conflict of interest.
