# Supplementary material for: The Development of the DDads Questionnaire: Awareness, Knowledge and Attitudes of the General Population Towards Paternal Depression
Source: Front Psychiatry. 2021 Jan 21;11:561954. doi: 10.3389/fpsyt.2020.561954 (PMC7859093; doi:10.3389/fpsyt.2020.561954)
Supplement: Supplementary file 1 [file Data_Sheet_1.PDF]

DUTCH (DELPHI ROUND 1)	ENGLISH (DELPHI ROUND 1)	DUTCH (CORRECTED AFTER DELPHI ROUND 1)	ENGLISH (CORRECTED AFTER DELPHI ROUND 1)	DUTCH (CORRECTED AFTER DELPHI ROUND 2)	ENGLISH (CORRECTED AFTER DELPHI ROUND 2)	DUTCH (CORRECTED AFTER INTERVIEWS LAY EXPERTS)	ENGLISH (CORRECTED AFTER INTERVIEWS LAY EXPERTS)
**ALGEMENE GEGEVENS PANELLID**	**GENERAL INFORMATION PANEL MEMBER**						
Jouw gegevens zijn enkel bekend bij de onderzoekers, worden anoniem verwerkt en worden vernietigd na verwerking.	*Your data are only known to the researchers, will be processed anonymously and destroyed after processing.*						
**Wat is je naam?**	**What is your name?**						
**Wat is je functie? (meerdere antwoordmogelijkheden mogelijk)**	**What is your function? (multiple answers possible)**						
☐ Psychiater<br>☐ Psycholoog<br>☐ Gynaecoloog<br>☐ Vroedvrouw/verpleegkundige werkzaam in de geestelijke gezondheidszorg<br>☐ Vroedvrouw werkzaam op een verloskamer/materniteit<br>☐ Eerstelijnsvroedvrouw/verpleegkundige<br>☐ Onderzoeker op een hogeschool/universiteit<br>☐ Docent in (para)medische opleiding (bvb. geneeskunde, vroedkunde, verpleegkunde)<br>☐ Andere (vrij in te vullen)	☐ Psychiatrist<br>☐ Psychologist<br>☐ Obstetrician<br>☐ Midwife / nurse working in mental health care<br>☐ Midwife working in a delivery room / maternity unit<br>☐ Primary care midwife / nurse<br>☐ Researcher at a university college / university<br><br>☐ Lecturer in (para) medical training (e.g. medicine, midwifery, nursing)<br>☐ Other (free to fill in)						
*Beoordeling relevantie en duidelijkheid vragen omtrent het "bewustzijn", de "kennis" en de "attitudes en overtuigingen" van paternale depressie<br>Gelieve aan te geven of je de onderdelen "bewustzijn", "kennis" en "attitudes en overtuigingen" van de vragenlijst relevant en duidelijk vindt.<br>Met **duidelijk** bedoelen we:<br>- moeilijke begrippen zijn vermeden en waar nodig voldoende uitgelegd<br>- vlot leesbaar en duidelijk<br>- duidelijk hoe de vraag ingevuld moet worden<br>- de antwoordmogelijkheden op de vragen zijn eenduidig (ze zijn niet op meerdere manieren uit te leggen)<br>Met **relevant** bedoelen we:<br>- draagt de vraag bij aan het antwoord op één van beide onderzoeksdoelen<br>- is de vraag en wijze van bevragen noodzakelijk om antwoord te krijgen op één van beide onderzoeksdoelen*	*Assessment of relevance and clarity about "awareness", the "knowledge" and the "attitudes and believes" about paternal depression. Please indicate if you feel that the components "awareness", "knowledge" and "attitudes and believes" of the questionnaire are relevant and clear.<br>By **clear** we mean:<br>- difficult concepts have been avoided and explained sufficiently where necessary<br>- easy to read and clear<br>- clear how the question should be filled in<br>- the answers to the questions are unambiguous (they cannot be explain in multiple ways)<br>By **relevant** we mean:<br>- does the question contributes to the answer on either of the research aims<br>- is the question and method of questioning necessary to get an answer on either of the research aims*						
**DEEL 1: BEWUSTZIJN**	**PART 1: AWARENESS**	**DEEL 1: BEWUSTZIJN**	**PART 1: AWARENESS**				
We peilen in dit deel naar het bewustzijn van de bevolking over mentale gezondheidsproblemen bij mannen gedurende de zwangerschap van hun partner én in de eerste 12 maanden na de geboorte.<br><br>Gelieve aan te geven of je de onderdelen in dit deel duidelijk en relevant vindt.	*In this section we are looking at the awareness of the population about mental health issues in men during their partners pregnancy and in the first 12 months after birth.<br>Please indicate if you feel that the components in this section are clear and relevant.*	*We peilen in dit deel naar het bewustzijn van de bevolking over psychische (mentale) gezondheidsproblemen bij mannen gedurende de zwangerschap van hun partner én in de eerste 12 maanden na de geboorte.*	*In this section we are looking at the awareness of the population about psychological (mental) health issues in men during their partners pregnancy and in the first 12 months after birth.*				
**Wat zijn volgens jouw de belangrijkste mentale gezondheidsproblemen die mannen ervaren gedurende de zwangerschap van hun partner? (open vraag - maximum 4 antwoorden)**	**What would you consider to be the main mental health problems in men during their partners' pregnancy? (open question - max. 4 responses)**	**Wat zijn volgens u de belangrijkste psychische (mentale) gezondheidsproblemen die mannen bij zichzelf ervaren gedurende de zwangerschap van hun partner? (open vraag - maximum 4 antwoorden)**	**What would you consider to be the main psychological (mental) health problems men experience with themselves during their partners' pregnancy? (open question - max. 4 responses)**				
In hoeverre was deze vraag duidelijk voor jou?<br>Likert schaal: Helemaal niet duidelijk - Eerder niet duidelijk - Eerder duidelijk - Helemaal duidelijk<br>Geef aan waarom.<br>In hoeverre was deze vraag relevant voor jou?<br>Likert schaal: Helemaal niet relevant - Eerder niet relevant - Eerder relevant - Helemaal relevant<br>Geef aan waarom.	*To what extent was this question was clear to you?<br>Likert scale: Not at all clear - Rather not clear - Rather clear - Very clear<br>Please explain why.<br>To what extent was this question relevant to you?<br>Likert scale: Not at all relevant - Rather not relevant - Rather relevant - Very relevant<br>Please explain why.*						
**Wat zijn volgens jou de belangrijkste mentale gezondheidsproblemen bij mannen in het eerste jaar na de geboorte? (open vraag - maximum 4 antwoorden)**	**In the first year after the baby is born, what do you consider to be the main mental health problems for men in this first year? (max. 4 responses)**	**Wat zijn volgens jou de belangrijkste psychische (mentale) gezondheidsproblemen ~~bij~~ die mannen bij zichzelf ervaren in het eerste jaar na de geboorte? (open vraag - maximum 4 antwoorden)**	**In the first year after the baby is born, what do you consider to be the main mental health problems men experience with themselves in this first year? (max. 4 responses)**				
In hoeverre was deze vraag duidelijk voor jou?<br>Helemaal niet duidelijk - Eerder niet duidelijk - Eerder duidelijk - Helemaal duidelijk<br>Geef aan waarom.<br>In hoeverre was deze vraag relevant voor jou?<br>Helemaal niet relevant - Eerder niet relevant - Eerder relevant - Helemaal relevant<br>Geef aan waarom.<br>Definitie paternale depressie: Paternale depressie wordt gedefinieerd als het voorkomen van een depressie of depressieve symptomen bij mannen in één van beide, de prenatale periode (voor de bevalling) of de postpartumperiode (tot 12 maanden na de bevalling)	*To what extent was this question was clear to you?<br>Not at all clear - Rather not clear - Rather clear - Very clear<br>Please explain why.<br>To what extent was this question relevant to you?<br>Not at all relevant - Rather not relevant - Rather relevant - Very relevant<br>Please explain why.<br><br>Definition paternal depression: Prenatal depression if defined as the occurrence of a depression or depressive symptoms in men in either the prenatal or postpartum period (first 12-months after childbirth).*						
**Hoe vaak komt paternale depressie voor bij mannen gedurende de zwangerschap van hun partner in de eerste 12 maanden na de geboorte? (% vrij in te vullen)**	**How common is paternal depression in men during their partners' pregnancy and in the first 12 months after childbirth? (% free to fill in)**						
In hoeverre was deze vraag duidelijk voor jou?<br>Helemaal niet duidelijk - Eerder niet duidelijk - Eerder duidelijk - Helemaal duidelijk<br>Geef aan waarom.<br>In hoeverre was deze vraag relevant voor jou?<br>Helemaal niet relevant - Eerder niet relevant - Eerder relevant - Helemaal relevant<br>Geef aan waarom.<br>Ontbreken er volgens jou items met betrekking tot het onderwerp paternale depressie die inzicht bieden in de onderzoeksvragen welke je toe zou voegen?<br>Heb je verder nog opmerkingen over de items in deel 1: bewustzijn?	*To what extent was this question was clear to you?<br>Not at all clear - Rather not clear - Rather clear - Very clear<br>Please explain why.<br>To what extent was this question relevant to you?<br>Not at all relevant - Rather not relevant - Rather relevant - Very relevant<br>Please explain why.<br>Do you think there are items missing on the subject of paternal depression that provide insights into the research questions you would add?<br>Do you have any comments about the items in part 1: awareness?*						
**DEEL 2: KENNIS VAN SYMPTOMEN, OORZAKEN EN BEHANDELINGEN**	**PART 2: KNOWLEDGE OF SYMPTOMS, CAUSES AND TREATMENTS**	**DEEL 2: KENNIS VAN SYMPTOMEN, OORZAKEN EN BEHANDELINGEN**	**PART 2: KNOWLEDGE OF SYMPTOMS, CAUSES AND TREATMENTS**	**DEEL 2: KENNIS VAN SYMPTOMEN EN BEHANDELINGEN**	**PART 2: KNOWLEDGE OF SYMPTOMS AND TREATMENTS**		
We peilen in dit deel naar de kennis van de bevolking over paternale depressie.<br><br>Gelieve aan te geven of je de onderdelen in dit deel duidelijk en relevant vindt.	*In this section we are looking at the knowledge of the population about paternal depression.<br>Please indicate if you feel that the components in this section are clear and relevant.*						
**Wat zijn oorzaken en symptomen van paternale depressie? (meerdere antwoorden mogelijk)**	**What are the causes and symptoms of paternal depression? (multiple answers possible)**						
☐ Slechte partnerrelatie: weinig affectiviteit, toename van relatieproblemen, partnergeweld.<br>☐ Negatief opvoedingsgedrag: zich verdrietig/neerslachtig/ellendig voelen (verminderde positieve emoties), verminderde affectie, verminderde gevoeligheid, toegenomen vijandigheid en verminderde betrokkenheid.<br>☐ Nieuwe persoonlijkheidskenmerken: terugtrekken/vermijden/isolatie (van sociale situaties, werk of gezin), besluiteloosheid, cynisme, woedeaanvallen, zelfkritiek, prikkelbaarheid.<br>☐ Oorzaken bij de partner: moeder had complicaties tijdens de zwangerschap/postpartum periode, verminderde affectie, aandacht en betrokkenheid in de relatie, verandering in seksuele relatie, tekort aan intimiteit, moeder is depressief.<br>☐ Oorzaken bij het kind: niet kunnen omgaan met de behoeften van het kind, het gevoel dat ze niet voldoende tijd hebben met hun kind.<br>☐ Sociale oorzaken: gebrek aan sociale steun, een beperkt gevoel van vrijheid, verslechtering van levensstijl. Gebruik van alcohol of andere drugs.<br>☐ Mentale oorzaken: onvoorbereid zijn op het ouderschap, stress/druk bijvoorbeeld zorgen over geldzaken, werk-privé balans, angst.<br>☐ Biologische oorzaken: hormonale veranderingen, vermoeidheid/slaapgebrek<br>☐ Andere:	☐ poor partner relationship: Affective rigidity, Increased marital conflict, Partner violence<br>☐ Negative parenting behaviour: Feeling sad/down/miserable (decreased positive emotions), decreased warmth, decreased sensitivity, increased hostility and disengagement<br>☐ New personal characteristics: Withdrawal/avoidance/isolation (from social situations, work, or family), Indecisiveness, Cynicism, Anger attacks, Self-criticism, Being irritable<br><br>☐ Partner causes: Mother had complications during pregnancy/postpartum period, Decline in affection, attention and closeness in their relationship, Change in sexual relationship, lack of intimacy, Mother is depressed<br>☐ Child causes: Not coping with infant's demands, Feeling they have not enough time with their infant<br>☐ Social causes: Lack of social support, A restricted sense of freedom, Deterioration of lifestyle, use of alcohol or other drugs<br>☐ Mental causes: Unprepared for parenthood, Stress/pressure e.g. worry about finances, work-life balance, Fear<br>☐ Biological causes: hormonal changes, Fatigue/lack of sleep<br>☐ Other:						
		**Wat zijn oorzaken van paternale depressie?**	**What are the causes of paternal depression?**				
		☐ Oorzaken bij de partner: moeder/partner had complicaties tijdens de zwangerschap/postpartum periode, verminderde affectie, aandacht en betrokkenheid in de relatie, verandering in seksuele relatie, tekort aan intimiteit, moeder/partner is depressief.<br>☐ Oorzaken bij het kind: niet kunnen omgaan met de behoeften van het kind, het gevoel dat ze niet voldoende tijd hebben met hun kind.<br>☐ Sociale oorzaken: gebrek aan sociale steun, een beperkt gevoel van vrijheid, ongezonde levensstijl.<br>☐ Mentale oorzaken: onvoorbereid zijn op het ouderschap, stress/druk bijvoorbeeld zorgen over geldzaken, werk-privé balans, angst.<br><br>☐ Biologische oorzaken: hormonale veranderingen, vermoeidheid/slaapgebrek	☐ Causes of the partner: mother/partner had complications during pregnancy/postpartum period, decreased affection, attention and involvement in the relationship, change in sexual relationship, lack of intimacy, mother/partner is depressed.<br>☐ Causes of the child: unable to deal with the needs of the child, feeling that they do not have enough time with their child.<br>☐ Social causes: lack of social support, a limited sense of freedom, unhealthy lifestyle.<br>☐ Mental causes: being unprepared for parenthood, stress/pressure for example concerns about money, work-private balance, fear.<br><br>☐ Biological causes: hormonal changes, fatigue/sleep deficiency				
		**Hoe kan je een paternale depressie herkennen (symptomen)?**	**How can you recognise a paternal depression (symptoms)?**	**Hoe kan je een paternale depressie herkennen (symptomen)?**			
		☐ Slechte partnerrelatie: weinig affectivieit, toename van relatieproblemen, partnergeweld.<br><br>☐ Negatief opvoedingsgedrag: zich verdrietig/neerslachtig/ellendig voelen (verminderde positieve emoties), verminderde affectie, verminderde gevoeligheid, toegenomen vijandigheid en verminderde betrokkenheid.<br><br>☐ Nieuwe persoonlijkheidskenmerken: terugtrekken/vermijden/isolatie (van sociale situaties, werk of gezin), besluiteloosheid, cynisme, woedeaanvallen, zelfkritiek, prikkelbaarheid.<br><br>☐ Lichamelijke symptomen: spijsverteringsproblemen, veranderingen in eetlust en gewicht, diarree, constipatie, hoofdpijn, tandpijn, misselijkheid en slapeloosheid<br>☐ Gebruik van alcohol of andere drugs.<br>*In hoeverre was deze vraag duidelijk voor jou?<br>Helemaal niet duidelijk - Eerder niet duidelijk - Eerder duidelijk - Helemaal duidelijk<br>Geef aan waarom.<br>In hoeverre was deze vraag relevant voor jou?<br>Helemaal niet relevant - Eerder niet relevant - Eerder relevant - Helemaal relevant<br>Geef aan waarom.*	☐ Poor partner relationship: low affection, increased relationship problems, partner violence.<br><br>☐ Negative upbringing behavior: feeling sad/depressed/miserable (reduced positive emotions), reduced affection, decreased sensitivity, increased hostility and reduced involvement.<br><br>☐ New personality characteristics: withdrawing/avoiding/isolation (from social situations, work or family), indecision, cynicism, raging attacks, self-criticism, irritability<br><br>☐ Somatic symptoms: digestive problems, changes in appetite and weight, diarrhea, constipation, headache, tooth pain, nausea and insomnia<br>☐ Use of alcohol or other drugs.<br>*To what extent was this question was clear to you?<br>Not at all clear - Rather not clear - Rather clear - Very clear<br>Please explain why.<br>To what extent was this question relevant to you?<br>Not at all relevant - Rather not relevant - Rather relevant - Very relevant<br>Please explain why.*	☐ Verandering van partnerrelatie: verminderde affectie, toename van relatieproblemen, partnergeweld.<br><br>☐ Negatief opvoedingsgedrag: zich verdrietig/neerslachtig/ellendig voelen (verminderde positieve emoties), verminderde affectie, verminderde gevoeligheid, toegenomen vijandigheid en verminderde betrokkenheid.<br><br>☐ Nieuwe persoonlijkheidskenmerken: terugtrekken/vermijden/isolatie (van sociale situaties, werk of gezin), besluiteloosheid, cynisme, woedeaanvallen, zelfkritiek, prikkelbaarheid.<br><br>☐ Lichamelijke symptomen: spijsverteringsproblemen, veranderingen in eetlust en gewicht, diarree, constipatie, hoofdpijn, tandpijn, misselijkheid en slapeloosheid<br>☐ Gebruik van alcohol en/of andere drugs.	☐ Change in partner relationship: low affection, increased relationship problems, partner violence.<br>☐ Negative upbringing behavior: feeling sad/depressed/miserable (reduced positive emotions), reduced affection, decreased sensitivity, increased hostility and reduced involvement.<br>☐ New personality characteristics: withdrawing/avoiding/isolation (from social situations, work or family), indecision, cynicism, raging attacks, self-criticism, irritability<br>☐ Somatic symptoms: digestive problems, changes in appetite and weight, diarrhea, constipation, headache, tooth pain, nausea and insomnia<br>☐ Use of alcohol and/or other drugs.		
**Welke types van behandeling zijn er geschikt voor mannen met een paternale depressie (meerdere antwoorden mogelijk)**	**What types of treatment are suitable for men experiencing paternal depression (multiple answers possible)**	**Welke types van behandeling zijn er geschikt voor mannen met een paternale depressie (meerdere antwoorden mogelijk)**	**What types of treatment are suitable for men experiencing paternal depression (multiple answers possible)**				

☐ Antidepressiva<br>☐ Meer socializen<br>☐ Ondersteuning van familie<br>☐ Praten en luisteren, met om het even wie<br>☐ Toetreden tot een steungroep<br>☐ Psychotherapie/counseling<br>☐ Ondersteuning van een vroedvrouw/verpleegkundige<br>☐ Hulp zoeken bij een huisarts<br>☐ Hulp zoeken bij een gynaecoloog<br>☐ Ondersteuning bij het huishouden<br>☐ Ondersteuning bij de kinderverzorging<br>☐ Trainen/sporten<br>☐ Rust/ontspanning/Tijd besteden aan zichzelf<br>☐ Evenwichtige voeding<br>☐ Alcohol<br>☐ Drugs<br><br>*In hoeverre was deze vraag duidelijk voor jou?*<br>*Helemaal niet duidelijk - Eerder niet duidelijk - Eerder duidelijk - Helemaal duidelijk*<br>*Geef aan waarom.*<br>*In hoeverre was deze vraag relevant voor jou?*<br>*Helemaal niet relevant - Eerder niet relevant - Eerder relevant - Helemaal relevant*<br>*Geef aan waarom.*	☐ Antidepressant medication<br>☐ Socialise more<br>☐ Family support<br>☐ Talking and listening, with anyone<br>☐ Join a support group<br>☐ Psychotherapy/counselling<br>☐ Support of a midwife/nurse<br>☐ Seeking help from a GP<br>☐ Seeking help from an obstetrician<br>☐ Household support<br>☐ Childcare support<br>☐ Exercise/sports<br>☐ Rest/relaxation/devote time to themselves<br>☐ Improved diet<br>☐ Alcohol<br>☐ Drugs<br><br>*To what extent was this question was clear to you?*<br>*Not at all clear - Rather not clear - Rather clear - Very clear*<br>*Please explain why.*<br>*To what extent was this question relevant to you?*<br>*Not at all relevant - Rather not relevant - Rather relevant - Very relevant*<br>*Please explain why.*	☐ Antidepressiva<br>☐ Onder de mensen komen<br>☐ Ondersteuning van familie<br>☐ Praten en luisteren, met om het even wie<br>☐ Toetreden tot een steungroep<br>☐ Ondersteuning van een vroedvrouw/verpleegkundige<br>☐ Hulp zoeken bij een huisarts<br>☐ Hulp zoeken bij een gynaecoloog<br>☐ Hulp zoeken bij een psycholoog<br>☐ Hulp zoeken bij een coach<br>☐ Ondersteuning bij het huishouden<br>☐ Ondersteuning bij de kinderverzorging<br>☐ Trainen/sporten<br>☐ Rust/ontspanning/Tijd besteden aan zichzelf<br>☐ Evenwichtige voeding<br>☐ Ziekteverlof<br>☐ Slapen	☐ Antidepressant medication<br>☐ See people<br>☐ Family support<br>☐ Talking and listening, with anyone<br>☐ Join a support group<br>☐ Support of a midwife/nurse<br>☐ Seeking help from a GP<br>☐ Seeking help from an obstetrician<br>☐ Seeking help from a psychologist<br>☐ Seeking help from a coach<br>☐ Household support<br>☐ Childcare support<br>☐ Exercise/sports<br>☐ Rest/relaxation/devote time to themselves<br>☐ Improved diet<br>☐ Sick leave<br>☐ Sleep				
**Als jij (of je man) een paternale depressie had, wie zou je eerste keuze zijn om naartoe te gaan (wie zou jij aanbevelen om als eerste naartoe te gaan? (Kies één)**	**If you (or your husband) had postnatal depression, who would be your first choice to go to? (who would you recommend they go to first? Pick one)**	**Als jij (of je man) een paternale depressie had, wie zou je eerste keuze zijn om naartoe te gaan (wie zou jij aanbevelen om als eerste naartoe te gaan? (Kies één)**	**If you (or your husband) had postnatal depression, who would be your first choice to go to? (who would you recommend they go to first? Pick one)**				
☐ Familie en vrienden<br>☐ Steungroep (online en offline)<br>☐ Vroedvrouw<br>☐ Verpleegkundige<br>☐ Huisarts<br>☐ Gynaecoloog<br>☐ Psycholoog<br>☐ Psychiater<br>☐ Zou geen hulp zoeken / aanbevelen<br>☐ Weet het niet<br>☐ Andere:<br><br>*In hoeverre was deze vraag duidelijk voor jou?*<br>*Helemaal niet duidelijk - Eerder niet duidelijk - Eerder duidelijk - Helemaal duidelijk*<br>*Geef aan waarom.*<br>*In hoeverre was deze vraag relevant voor jou?*<br>*Helemaal niet relevant - Eerder niet relevant - Eerder relevant - Helemaal relevant*<br>*Geef aan waarom.*<br>*Ontbreken er volgens jou items met betrekking tot het onderwerp paternale depressie die inzicht bieden in de onderzoeksvragen welke u toe zou voegen?*<br>*Heb je verder nog opmerkingen over de items in deel 2: kennis van symptomen oorzaken en behandelingen?*	☐ Family and friends<br>☐ Support group (online and offline)<br>☐ Midwife<br>☐ Nurse<br>☐ GP<br>☐ Obstetrician<br>☐ Psychologist<br>☐ Psychiatrist<br>☐ Would not seek help<br>☐ Do not know<br>☐ Other<br><br>*To what extent was this question was clear to you?*<br>*Not at all clear - Rather not clear - Rather clear - Very clear*<br>*Please explain why.*<br>*To what extent was this question relevant to you?*<br>*Not at all relevant - Rather not relevant - Rather relevant - Very relevant*<br>*Please explain why.*<br>*Do you think there are items missing on the subject of paternal depression that provide insights into the research questions you would add?*<br>*Do you have any comments about the items in part 2: knowledge of symptoms, causes and treatments?*	☐ Familie of en vrienden<br>☐ Steungroep (online en offline)<br>☐ Coach<br>☐ Vroedvrouw<br>☐ Verpleegkundige<br>☐ Huisarts<br>☐ Gynaecoloog<br>☐ Psycholoog<br>☐ Psychiater<br>☐ Zou geen hulp zoeken / aanbevelen.<br>☐ Weet het niet<br>☐ Andere:	☐ Family or and friends<br>☐ Support group (online and offline)<br>☐ Coach<br>☐ Midwife<br>☐ Nurse<br>☐ GP<br>☐ Obstetrician<br>☐ Psychologist<br>☐ Psychiatrist<br>☐ Would not seek help<br>☐ Do not know<br>☐ Other				
**DEEL 3: ATTITUDES EN OVERTUIGINGEN**	**PART 3: ATTITUDES AND BELIEFS**	**DEEL 3: ATTITUDES EN OVERTUIGINGEN**	**PART 3: ATTITUDES AND BELIEFS**				
We peilen in dit deel naar de houding en opvattingen van de bevolking over paternale depressie.<br>*Het doel van dit deel is om te peilen de attitudes en overtuigingen van de bevolking over paternale depressie. Gelieve aan te geven of je de onderdelen in dit deel duidelijk en relevant vindt.*	*In this section we are looking at the attitudes and believes of the population about paternal depression.*<br>*The aim of this section is to look at the attitudes and believes of the population about paternal depression. Please indicate if you feel that the components in this section are clear and relevant.*						
**Geef aan in hoeverre ben je het eens bent met volgende stellingen (Likert schaal: Helemaal niet akkoord - Niet akkoord - Eerder niet akkoord - Eerder akkoord - Akkoord - Helemaal akkoord - Weet het niet):**	**Indicate to what extent you agree with the following statements (Likert scale: Strongly disagree - Disagree - Slightly disagree - Slightly agree - Agree - Strongly agree - Don't know):**						
☐ Het is normaal dat mannen zich depressief voelen tijdens de zwangerschap van hun partner.<br>☐ Paternale depressie is een normaal onderdeel van het ouderschap.<br>☐ Weten hoe je voor een baby moet zorgen is vanzelfsprekend voor mannen.<br>☐ Mannen krijgen een paternale depressie omdat ze het ouderschap niet aankunnen.<br>☐ Mannen krijgen een paternale depresssie omdat ze onrealistische verwachtingen hebben.<br>☐ Paternale depressie bestond niet in vorige generaties.<br>☐ Paternale depressie is niet ernstig.<br>☐ Paternale depressie vereist een specifieke behandeling.<br>☐ Paternale depressie zal vanzelf verdwijnen als de baby ouder wordt.<br>☐ We spreken alleen van een paternale depressie als je het kind wil kwetsen of vermoorden.<br>☐ We spreken alleen van paternale depressie als je aan zelfmoord denkt.<br>☐ Alleen carrière mannen krijgen een paternale depressie.<br>☐ Mannen van wie de partner depressief is, hebben een verhoogd risico op een depressie.<br>☐ Alle mannnen zouden moeten gecontroleerd worden op depressie tijdens de zwangerschap.<br>☐ Alle mannnen zouden moeten gecontroleerd worden op depressie nadat de baby geboren is.<br>☐ Mannen zouden alleen gecontroleerd moeten worden op depressie als hun partner depressief is.<br>☐ Mannen die medicatie nemen voor paternale depressie zijn zwak.<br>☐ Mannen met een paternale depressie kunnen geen goede vaders zijn.<br>☐ Paternale depressie is een teken van zwakte.<br>☐ Mannen kiezen zelf voor een paternale depressie.<br><br>*In hoeverre was deze vraag duidelijk voor jou?*<br>*Helemaal niet duidelijk - Eerder niet duidelijk - Eerder duidelijk - Helemaal duidelijk*<br>*Geef aan waarom.*<br>*In hoeverre was deze vraag relevant voor jou?*<br>*Helemaal niet relevant - Eerder niet relevant - Eerder relevant - Helemaal relevant*<br>*Geef aan waarom.*<br>*Ontbreken er volgens jou items met betrekking tot het onderwerp paternale depressie die inzicht bieden in de onderzoeksvragen welke je toe zou voegen?*<br><br>*Heb je verder nog opmerkingen over de items in deel 3: attitudes en overtuigingen?*	☐ It is normal for men to feel depressed during his partner’s pregnancy.<br>☐ Paternal depression is a normal part of becoming a parent.<br>☐ Knowing how to look after a baby comes naturally to men.<br>☐ Men get perinatal depression because they can not cope with parenthood.<br>☐ Men get perinatal depression because they have unrealistic expectations.<br>☐ Paternal depression did not exist in previous generations<br>☐ Paternal depression is not serious.<br>☐ Paternal depression requires special treatment.<br>☐ Paternal depression will go away on its own as the baby gets older.<br>☐ It is only paternal depression when you want to harm or kill the child.<br>☐ It is only paternal depression when you’re thinking about suicide.<br>☐ Only career men get perinatal depression.<br>☐ Men whose partners are depressed are also at risk for depression.<br>☐ All men should be checked for depression during pregnancy.<br>☐ All men should be checked for depression after the baby is born.<br>☐ Men should only be checked for depression when their partner is depressed.<br>☐ Men who take medication for paternal depression are weak-willed.<br>☐ Men with postnatal depression can’t be good fathers<br>☐ Paternal depression is a sign of weakness.<br>☐ Men choose to get paternal depression.<br><br>*To what extent was this question was clear to you?*<br>*Not at all clear - Rather not clear - Rather clear - Very clear*<br>*Please explain why.*<br>*To what extent was this question relevant to you?*<br>*Not at all relevant - Rather not relevant - Rather relevant - Very relevant*<br>*Please explain why.*<br>*Do you think there are items missing on the subject of paternal depression that provide insights into the research questions you would add?*<br><br>*Do you have any comments about the items in part 3: attitudes and beliefs?*	☐ Het is normaal dat mannen zich depressief voelen tijdens de zwangerschap van hun partner.<br>☐ Paternale depressie is een normaal onderdeel van het ouderschap.<br>☐ Weten hoe je voor een baby moet zorgen is vanzelfsprekend voor mannen.<br>☐ Mannen krijgen een paternale depressie omdat ze het ouderschap niet aankunnen.<br>☐ Mannen krijgen een paternale depresssie omdat ze onrealistische verwachtingen hebben.<br>☐ Paternale depressie bestond niet in vorige generaties.<br>☐ Paternale depressie is niet ernstig.<br>☐ Paternale depressie vereist een specifieke behandeling.<br>☐ Paternale depressie zal vanzelf verdwijnen als de baby ouder wordt.<br>☐ We spreken alleen van een paternale depressie als je het kind wil kwetsen of vermoorden.<br>☐ We spreken alleen van paternale depressie als je aan zelfmoord denkt.<br>☐ Alleen carrière mannen krijgen een paternale depressie.<br>☐ Mannen van wie de partner depressief is, hebben een verhoogd risico op een depressie.<br>☐ Alle mannnen zouden moeten gecontroleerd worden op depressie tijdens de zwangerschap.<br>☐ Alle mannnen zouden moeten gecontroleerd worden op depressie nadat de baby geboren is.<br>☐ Mannen zouden alleen gecontroleerd moeten worden op depressie als hun partner depressief is.<br>☐ Mannen die medicatie nemen voor paternale depressie zijn zwak.<br>☐ Mannen met een paternale depressie kunnen geen goede vaders zijn.<br>☐ Paternale depressie is een teken van zwakte.<br>☐ Mannen kiezen zelf voor een paternale depressie.<br><br>☐ Mannen met een paternale depressie willen gewoon aandacht van hun partner.	☐ It is normal for men to feel depressed during his partner’s pregnancy.<br>☐ Paternal depression is a normal part of becoming a parent.<br>☐ Knowing how to look after a baby comes naturally to men.<br>☐ Men get perinatal depression because they can not cope with parenthood.<br>☐ Men get perinatal depression because they have unrealistic expectations.<br>☐ Paternal depression did not exist in previous generations<br>☐ Paternal depression is not serious.<br>☐ Paternal depression requires special treatment.<br>☐ Paternal depression will go away on its own as the baby gets older.<br>☐ It is only paternal depression when you want to harm or kill the child.<br>☐ It is only paternal depression when you’re thinking about suicide.<br>☐ Only career men get perinatal depression.<br>☐ Men whose partners are depressed are also at risk for depression.<br>☐ All men should be checked for depression during pregnancy.<br>☐ All men should be checked for depression after the baby is born.<br>☐ Men should only be checked for depression when their partner is depressed.<br>☐ Men who take medication for paternal depression are weak-willed.<br>☐ Men with postnatal depression can’t be good fathers<br>☐ Paternal depression is a sign of weakness.<br>☐ Men choose to get paternal depression.<br><br>☐ Men with paternal depression just want their partner's attention.				
**DEEL 4: PERSOONSGEGEVENS**	**PART 4: DEMOGRAPHIC DATA**	**DEEL 4: PERSOONSGEGEVENS**	**PART 4: DEMOGRAPHIC DATA**				
In het vierde en ook het laatste deel worden jouw persoonsgegevens bevraagd, we vragen hiernaar zodat we de resultaten van het onderzoek beter kunnen interpreteren (slechts één antwoord mogelijk, kruis het vakje aan en/of vul in).<br>*Het doel van deze vragen is om na te gaan of er een relatie is tussen socio-demografische karakteristieken van de bevolking en hun bewustzijn, kennis en attitudes omtrent paternale depressie.*	*In the fourth and last section we ask for your personal information, to better understand the results of the survey (only one answer possible, tick the box and/or complete.*<br>*The aim of these questions is to assess the relationship between socio-demographic characteristics of the population and their awareness, knowledge and attitudes about paternal depression.*						
**Wat is je leeftijd?**	**What is your age?**						
☐18-25 jaar<br>☐26-35 jaar<br>☐36-45 jaar<br>☐46-55 jaar<br>☐56-65 jaar<br>☐66-75 jaar<br>☐75 jaar of ouder	☐18-25 year<br>☐26-35 year<br>☐36-45 year<br>☐46-55 year<br>☐56-65 year<br>☐66-75 year<br>☐75 year of older						
**Wat is je geslacht?**	**What is your gender?**						
☐Man<br>☐Vrouw	☐Male<br>☐Female						
**Wat is je burgerlijke staat?**	**What is your marital status?**						
☐Getrouwd<br>☐Samenwonend<br>☐Alleenstaand of in een latrelatie	☐Married<br>☐Cohabiting<br>☐Single or long distance relationship						
**Heb je kinderen?**	**Do you have children?**	**Heb je kinderen?**	**Do you have children?**				
☐Ja<br>☐Neen	☐Yes<br>☐No	☐Ja<br>Hoeveel?<br>☐Neen	☐Yes<br>How many?<br>☐No				
**Wat is je hoogste opleiding waarvoor je een diploma hebt gehaald?**	**What is your highest education for which you have obtained a diploma?**						
☐Geen<br>☐Lagere school<br>☐Secundaire school<br>☐Hoger onderwijs (niet universitair)<br>☐Universitair onderwijs<br>☐Andere:	☐No education<br>☐Primary level<br>☐Secondary level<br>☐Tertiary education (not university)<br>☐Tertiary education (university)<br>☐Other:						
**Wat is je hoofdberoep**	**What is your main professional occupation?**	**Heb je een eigen inkomen?**	**Do you have your own income?**				

☐In loondienst<br>☐Zelfstandige<br>☐Werkloos<br>☐Student<br>☐Gepensioneerd<br>Postcode (vrij veld)	☐Employed<br>☐Self-Employed<br>☐Unemployed<br>☐Student<br>☐Retired<br>Postal code (free space)	☐Loontrekkende<br>☐Zelfstandige<br>☐Steuntrekkende of vervangingsinkomen<br>☐Geen inkomen<br>☐Gepensioneerd<br><br>Postcode (vrij veld)	☐Employed<br>☐Self-Employed<br>☐Welfare of Replacement income<br>☐No income<br>☐Student<br>☐Retired<br>Postal code (free space)				
Welke taal is je moedertaal (Het gaat erom welke taal je het meest spreekt. Dit kan meer dan één zijn. Je moet dan wel alle aangeduide talen vloeiend spreken.)?	Which language is your mother tongue (It is about which language you speak most. This can be more than one. You have to speak all the indicated languages fluently.)?						
☐Nederlands<br>☐Frans<br>☐Engels<br>☐Arabisch<br>☐Andere	☐Dutch<br>☐French<br>☐English<br>☐Arabic<br>☐Other						
Heb je een opleiding gevolgd in de mentale gezondheidszorg?	Did you have any training in the field of mental health?						
☐Ja<br>☐Neen	☐Yes<br>☐No						
**Heb je ooit mentale gezondheidsproblemen gehad?**	**Have you ever had mental health problems?**	**Heb je ooit professionele hulp gezocht voor mentale gezondheidsproblemen?** ***Mentale gezondheidsproblemen variëren van veelvoorkomende problemen, zoals depressie en angstaanvallen, tot zeldzamere problemen zoals schizofrenie en bipolaire stoornis (manisch-depressieve stoornis).***	**Have you ever sought professional help for mental health problems? Mental health problems vary from common problems such as depression and anxiety attacks, to uncommon problems such as schizophrenia and bipolar disorder (manic-depressive disorder).**				
☐Ja<br>☐Depressie gerelateerd aan de perinatale periode (gedurende de eerste 12 maanden na de geboorte van je kind)<br>☐Depressie niet gerelateerd aan de perinatale periode (gedurende de eerste 12 maanden na de geboorte van je kind)<br>☐Andere<br>☐Neen	☐Yes<br>☐Depression related to the perinatal period (during the first 12 months after the birth of your child)<br>☐Depression not related to the perinatal period (during the first 12 months after the birth of your child)<br>☐Other<br>☐No.	☐Ja<br>☐Depressie gerelateerd aan de perinatale periode (gedurende de eerste 12 maanden na de geboorte van je kind)<br>☐Depressie niet gerelateerd aan de perinatale periode (gedurende de eerste 12 maanden na de geboorte van je kind)<br>☐Andere<br>☐Neen	☐Yes<br>☐Depression related to the perinatal period (during the first 12 months after the birth of your child)<br>☐Depression not related to the perinatal period (during the first 12 months after the birth of your child)<br>☐Other<br>☐No.				
**Had je voor het invullen van deze vragenlijst al ooit gehoord van paternale depressie?**	**Did you ever hear of paternal depression before completing this questionnaire?**	**Had je voor het invullen van deze vragenlijst al ooit gehoord van paternale depressie?**	**Did you ever hear of paternal depression before completing this questionnaire?**				
☐Ja<br>☐Studies/werk<br>☐Media<br>☐Vrienden en familie<br>☐Andere:<br>☐Neen<br>*In hoeverre waren deze vragen duidelijk voor jou?*<br>*Likert schaal: Helemaal niet duidelijk - Eerder niet duidelijk - Eerder duidelijk - Helemaal duidelijk*<br>*Geef aan waarom je deze vragen helemaal niet / eerder niet duidelijk vindt.*<br>*In hoeverre waren deze vragen relevant voor jou?*<br>*Likert schaal: Helemaal niet relevant - Eerder niet relevant - Eerder relevant - Helemaal relevant*<br>*Geef aan waarom je deze vragen helemaal niet / eerder niet relevant vindt.*	☐Yes<br>☐Studies/work<br>☐Media<br>☐Friends and family<br>☐Other:<br>☐No<br><br>*To what extent were these questions was clear to you?*<br>*Likert scale: Not at all clear - Rather not clear - Rather clear - Very clear*<br>*Please explain why.*<br>*To what extent were these questions was relevant to you?*<br>*Likert scale: Not at all relevant - Rather not relevant - Rather relevant - Very relevant*<br>*Please explain why.*	☐Ja<br>☐Studies/werk<br>☐Media<br>☐Vrienden or familie<br>☐Andere:<br>☐Neen	☐Yes<br>☐Studies/work<br>☐Media<br>☐Friends or family<br>☐Other:<br>☐No				
**Stelling: Het opzet van de vragenlijst is volgens jou een correcte manier om een antwoord te krijgen op de onderzoeksvragen?**	**Statement: The format of the questionnaire is in your opinion a correct way to get an answer to the research questions?**						
**Tot slot: Indien je nog algemene vragen, suggesties of opmerkingen heeft kan je die hier kwijt.**	**Finally: If you have any questions, suggestions or comments, please let us know here.**						
