# Supplementary material for: The Development of the DDads Questionnaire: Awareness, Knowledge and Attitudes of the General Population Towards Paternal Depression
Source: Front Psychiatry. 2021 Jan 21;11:561954. doi: 10.3389/fpsyt.2020.561954 (PMC7859093; doi:10.3389/fpsyt.2020.561954)
Supplement: Supplementary file 2 [file Data_Sheet_2.PDF]

Some open-ended/qualitative feedback on the questionnaire of content experts.

Question	Open-ended/qualitative feedback on the questionnaire of content experts in round 1	Question (English)	Open-ended/qualitative feedback on the questionnaire of content experts in round 1 (English)
Wat zijn oorzaken van paternale depressie- Geef aan waarom u dit helemaal niet / eerder niet relevant vindt.	/	What are the causes and symptoms of paternal depression? Please indicate why you find this items is not at all/ rather not relevant	/
Wat zijn oorzaken van paternale depressie- Geef aan waarom u waarom u niet 'helemaal relevant' heeft aangeduid-	ik vind de opsomming na elke oorzaak soms erg ruim....je herkent je mogelijks wel altijd ergens in...	What are the causes and symptoms of paternal depression? Please indicate why you did not indicate this items is very relevant	I think that the list after each cause is very widely … you may always recognise yourself …
	Belangrijk om ook de herbelevingen uit eigen verleden van de partner niet te vergeten		Important not to forget relivings from the past of the partner
	eerder wel relevant aangeduid...		rather relevant indicated
Wat zijn oorzaken van paternale depressie- Geef aan waarom u dit helemaal niet / eerder niet duidelijk vindt.	onvolledige antwoorden: eerste antwoord "complicaties bij de moeder": er wordt enkel gesproken over psychische symptomen waarvan een aantal niet "complicaties" zijn, maar eerder een realiteit. anderzijds worden échte medische complicaties niet vermeld (wond-infectie, ernstig bloedverlies, ecclampsie...) die mogelijk ook aanleiding kunnen zijn tot paternale depressie.	What are the causes and symptoms of paternal depression? Please indicate why you find this items is not at all/ rather not clear	incomplete answers: first answer "complications in the mother": one speaks only about mental symptoms, not all are "complications", but rather reality. on the other hand real medical complications are not mentioned (wound-infection, severe blood loss, eclampsia...) which also can be lead to paternal depression.

Ook bij oorzaken bij kind: enkel aandacht voor de psychologische redenen, maar bv handicap of medische complicaties, vroeggeboorte etc worden niet vermeld.	Also by the causes of the child: only attention for the psychological causes, but for instance disability of medical complications, prematurity etc are not mentioned
In de eerste bulletpoint staat nog moederpartner (dit is niet correct den kik)	In the first bullet point still motherpartner is mentioned (that is not correct I guess)
Omdat	Because
bij de eerste bullet het woord moederpartner mijn mij vragen oproep	At the first bullet point the word motherpartner raises questions
bij de tweede bullet: moeten beide ouders, 1 van beide of geen van beide kunnen omgaan met d ebehoeften van het kind	at the second bullet: should both parents, one of both or either one of them be able to cope with the needs of the child
derde: wie ervaart dit, vader of moeder of beide	third: who experiences this, father or mother or both
meer dan een keuze moet mogelijk zijn	more than one option should be possible
ik vond het woord moederpartner verwarrend. Ik dacht spontaan aan de moeder van de partner, maar ik denk dat jullie gewoon partner bedoelen. Al dan niet moeder of vader.	I think that the word motherpartner was confusing. Spontaneously I thought on the mother of the partner, but I think you just mean the partner. Whether or not mother or father.
Het woord 'moederpartner' geeft verwarring (gaat het over de partner of moeder van de partner (dus schoonmoeder?). Ik zou verder de term 'paternale depressie' goed duiden. Weet de doorsnee bevolking wat we bedoelen met paternale depressie...	The word 'motherpartner' is confusing (do you mean the partner or mother of the partner, so mother-in-law?). Further I would indicate the term 'paternal depression' sufficiently. Does the general population know what we mean by paternal depression …

	'term 'verslechtering van levenstijl' is onvoldoende duidelijk. Bij biologische oorzaken weet ik niet zo gauw wat dan hormonale veranderingen teweegbrent bij de man. Associeer je steeds met een vrouw. Dus deze term is naar mijn gevoel ook niet duidelijk genoeg.		term 'deterioration of life style' is not clear enough. by biological causes I don't know what then causes hormonal changes to the man. You always associate that with a woman. So this term is in my opinion not clear enough.
Wat zijn oorzaken van paternale depressie- Geef aan waarom u waarom u niet 'helemaal duidelijk' heeft aangeduid-	Ik weet niet in hoeverre 'verminderde affectie' voor iedereen voldoende duidelijk is.	What are the causes and symptoms of paternal depression? Please indicate why you did not indicate this items is very clear	I don't know to what extent 'deceased affection' is clear to everyone
	moederpartner is een niet bestaand woord		motherpartner is a non-existent word
	omdat er meerdere voorbeelden kunnen gelden en dit niet meteen duidelijk is dat het voorbeelden zijn		because several examples may be applicable and it is not immediately clear that these are examples
	'moederpartner' is voor mij een ongekende term, klinkt wat vreemd		motherpartner" in an unknown term to me, sounds somehow weird
